# Thymoquinone decreases F-actin polymerization and the proliferation of human multiple myeloma cells by suppressing STAT3 phosphorylation and Bcl2/Bcl-_XL _expression

**DOI:** 10.1186/1476-511X-10-236

**Published:** 2011-12-16

**Authors:** Gamal Badr, Mohamed Mohany, Faisal Abu-Tarboush

**Affiliations:** 1 Zoology Department, College of Science, King Saud University, Riyadh, Saudi Arabia; 2 Deanship of Scientific research, King Saud University, Riyadh, Saudi Arabia; 3 Zoology Department, Faculty of Science, Assiut University, 71516 Assiut, Egypt

**Keywords:** Cytoskeleton, multiple myeloma, proliferation, signaling, thymoquinone

## Abstract

**Background:**

Thymoquinone (TQ), the major active component of the medicinal herb Nigella sativa Linn., has been described as a chemopreventive and chemotherapeutic compound.

**Methods:**

In this study, we investigated the effect of TQ on survival, actin cytoskeletal reorganization, proliferation and signal transduction in multiple myeloma (MM) cells.

**Results:**

We found that TQ induces growth arrest in both MDN and XG2 cells in a dose- and time-dependent manner. TQ also inhibited CXC ligand-12 (CXCL-12)-mediated actin polymerization and cellular proliferation, as shown by flow cytometry. The signal transducer and activator of transcription (STAT) and B-cell lymphoma-2 (Bcl-2) signaling pathways may play important roles in the malignant transformation of a number of human malignancies. The constitutive activation of the STAT3 and Bcl-2 pathways is frequently observed in several cancer cell lines, including MM cells. Using flow cytometry, we found that TQ markedly decreased STAT3 phosphorylation and Bcl-2 and Bcl-_XL _expression without modulating STAT5 phosphorylation in MM cells. Using western blotting, we confirmed the inhibitory effect of TQ on STAT3 phosphorylation and Bcl-2 and Bcl-_XL _expression.

**Conclusions:**

Taken together, our data suggests that TQ could potentially be applied toward the treatment of MM and other malignancies.

## Background

Multiple myeloma (MM) is a monoclonal plasma cell malignancy that, despite intensive investigation, remains universally fatal [1]. Clinically, it is characterized by high levels of paraproteins in the blood and/or urine, lytic bone lesions, anemia, renal dysfunction, and bone marrow (BM) plasmacytosis [2]. Current treatments for this disease include combination chemotherapy with or without stem cell transplantation and the use of alkylating agents, glucocorticosteroids, and thalidomide [3]. Therefore, there is a need to further identify the factors and mechanisms responsible for maintaining the survival and proliferation of MM cells and mediating tumorigenesis and drug resistance. Previous studies have reported that MM cell proliferation is a prognostic factor and is associated with angiogenesis [4]. The actin cytoskeleton and its regulatory proteins are crucial for the migration of most cells. During cell migration, the actin cytoskeleton is dynamically remodeled, which produces the force necessary for cell migration [5]. Because inhibiting these processes decreases cell motility, elucidating the molecular mechanisms of actin reorganization is important for cancer therapeutics. Chemokines, such as CXCL-12, and its receptor CXCR4, have been shown to be involved in the homing and migration of MM cells by attracting and activating plasma cells in the BM [6].

The signal transducer and activator of transcription (STAT) family of transcription factors is associated with inflammation and the survival, proliferation, metastasis, angiogenesis, and chemoresistance of tumor cells [7]. The STAT3 and STAT5 transcription factors have been implicated in numerous malignancies. They are the final targets of IL-6 and IGF-1, respectively, and can stimulate the growth of B cells [8]. STAT3 is constitutively expressed in MM, leukemia, lymphoma, squamous cell carcinoma, and other solid tumors, including cancers of the prostate, breast, head and neck, and nasopharynx [7]. STAT3 may also promote tumor cell transformation via anti-apoptotic signaling (i.e., by up-regulating the genes that counteract active cell death). In cell lines from brain, skin, and breast tumors, the overexpression of anti-apoptotic genes, such as Bcl-2 and Bcl-_XL_, is associated with constitutive STAT3 activity, and STAT3 inhibition induces apoptosis [9-11]. The inhibition of signal mediators acting upstream of STAT3 and the use of dominant-negative variants of STAT3 have been shown to reduce proliferation or enhance apoptosis in various cell types [12,13].

Thymoquinone (TQ) is considered to be the bioactive and the most abundant constituent of volatile black seed oil and has been shown to have anti-inflammatory and antioxidant effects [14]. In particular, Shoieb et al. [2003] found *in vitro *inhibition of growth and induction of apoptosis in cancer cell lines in response to TQ [15]. In addition, TQ has the ability to kill several types of tumors without significant toxicity to normal cells, indicating that this compound may be a potential chemotherapeutic agent [16]. Recently, Ravindran et al. [2010] published a detailed study of the anti-proliferative, anti-inflammatory and chemosensitization activities of TQ in myeloid leukemia cells [17]. Although most of their data focused on myeloid leukemia cells, they compared the effect of TQ on cells from various types of cancers. Furthermore, it has been reported that TQ inhibits proliferation, induces apoptosis and chemosensitizes human MM cells by suppressing the signal transducer and activator of the transcription 3 activation pathway [18]. Thus, TQ exerts an anti-neoplastic effect and may be a promising dietary chemopreventive agent.

Although TQ modulates proliferation and induces growth arrest in numerous cancer cells, few data are available concerning its effect on MM cells. Therefore, in this study, we investigated the effect of TQ on survival, actin cytoskeletal reorganization, proliferation and signal transduction in MM cells.

## Materials and methods

### Cell culture and reagents

MDN and XG-2 cells are IL-6-dependent human myeloma cells [19,20]. These MM cells were maintained in RPMI 1640 medium containing 10 mM HEPES, 10% fetal calf serum (FCS; BioWhittaker, Walkersville, MD, USA) and 1% L-glutamate (R-10 medium) supplemented with 3 ng/mL IL-6 (Peprotech, Rocky Hill, NJ, USA). The cell cultures were free of *Mycoplasma*, as assessed by an enzyme-linked immunosorbent assay (ELISA) kit (Boehringer, Mannheim, Germany). All aspects of this study were approved by ethical committee according to the Declaration of Helsinki.

### Anti-proliferation assay

The anti-proliferative effect of TQ on MDN and XG2 cells was determined using the 3-(4,5-dimethylthiazol-2-yl)-2,5-diphenyltetrazolium bromide (MTT) uptake method. Briefly, 5 × 10^4 ^cells/well were plated on 48-well culture plates and allowed to adhere at 37°C for 12 h. The following day, various doses of TQ were added to the cells, which were further incubated for 12 h. Next, MTT (0.5 mg) was added to 1 mL of the cell suspension for 4 h. The ability of the cells to form formazan crystals via active mitochondrial respiration was determined using a microplate reader (Titertek Multiskan, Flow Laboratories, North Ryde, Austria) after dissolving the crystals in DMSO. An empty well was used as a blank. Cytotoxicity was expressed as the relative percentage of the absorbance measured in the control and TQ-treated cells. Morphological changes after exposure to the TQ extract were observed under a phase-contrast inverse microscope (Olympus, Japan).

### F-actin polymerization assay

MM cells were cultured for 24 h in culture medium with or without TQ. Intracellular F-actin polymerization was assessed as previously described [21]. Briefly, the cells were harvested and resuspended (4 × 10^6^/ml) in HEPES-buffered RPMI 1640 at 37°C with or without CXCL-12 (250 ng/ml). At the indicated times, the cells (100 μl) were added to 400 μl of assay buffer containing 4 × 10^-7 ^M FITC-labeled phalloidin, 0.5 mg/ml L-α-lysophosphatidylcholine (both from Sigma-Aldrich) and 4.5% formaldehyde in PBS. The fixed cells were analyzed using flow cytometry, and the mean fluorescence intensity (MFI) was determined for each sample. The percentage change in the MFI was calculated for each sample at each time point using the following formula: (1-(MFI before addition of CXCL-12/MFI after addition of CXCL-12) × 100.

### CFSE proliferation assays

MM cells were harvested, washed twice in PBS and stained with 0.63 μM carboxyfluorescein diacetate succinimidyl ester (CFSE) (Molecular Probes, Eugene, OR, USA) for 8 min at room temperature. The residual CFSE was removed by washing three times in PBS, and the CFSE-labeled cells were seeded in 6-well plates. The cells were then grown for 5 days in cell culture medium with or without TQ (10 μM). The CFSE fluorescence intensity was measured using FACS.

### Intracellular phospho-specific flow cytometry

MM cells (with or without TQ treatment) were fixed for 10 minutes in pre-warmed cytofix buffer (BD Cytofix #554655). The cells were permeabilized for 30 minutes on ice in PERM III buffer (BD PERM-III buffer #558050). The permeabilized cells were washed twice, re-suspended in staining buffer (PBS plus 0.5% bovine serum albumin), and stained in a final volume of 100 μL for 30 min at room temperature. STAT3 and STAT5 phosphorylation, Bcl-2 and Bcl-_XL _expression and control IgG quantification were assessed in the cells using the Phosflow method (BD Biosciences), according to the manufacturer's instructions. The cells were fixed and directly analyzed using a FACSCalibur (BD Pharmingen).

### Immunoblotting

Whole-cell lysates were prepared from TQ-treated and untreated cells in RIPA buffer (20 mM Tris-HCl, pH 7.5, 120 mM NaCl, 1.0% Triton 6100, 0.1% SDS, 1% sodium deoxycholate, 10% glycerol, 1 mM EDTA and 1% protease inhibitor cocktail (Roche)). Following centrifugation at 16,000 × *g *at 4°C for 15 min, the protein concentrations in the supernatants were determined using a protein assay kit (Bio-Rad, Hercules, CA, USA). Equal amounts of protein from the whole-cell lysates were mixed with reducing sample buffer (0.92 M Tris-HCl, pH 8.8, 1.5% SDS, 4% glycerol, and 280 mM 2-ME) and separated using discontinuous SDS-PAGE. The proteins were transferred onto nitrocellulose membranes using a Bio-Rad Trans-Blot electrophoretic transfer device and blocked for 1 h at room temperature with 1% BSA or 5% skim milk dissolved in TBS (20 mM Tris-HCl, pH 7.4, 150 mM NaCl) supplemented with 0.1% Tween 20. The membranes were then incubated in the same blocking buffer with rabbit polyclonal antibodies to STAT3 and STAT5 and mouse monoclonal antibodies against phosphorylated STAT3 (Tyr705) and phosphorylated STAT5, Bcl-2 and Bcl-_XL _(1:1,000; all from Santa Cruz Biotechnology). The blots were rinsed thoroughly and then incubated with an HRP-labeled species-matched secondary antibody for 1 h. The protein bands were detected using enhanced chemiluminescence reagents (ECL, SuperSignal West Pico Chemiluminescent Substrate, Perbio, Bezons, France), and the ECL signals were visualized using hyperfilm ECL. To quantify the band intensities, the films were scanned, saved as TIFF files and analyzed using NIH ImageJ. The percentages of STAT3 and STAT5 phosphorylation, as well as the percentages of Bcl-2 and Bcl-_XL _expression were calculated as the intensities of protein bands relevant to the corresponding actin bands × 100.

### Statistical analyses

The data were analyzed using the SPSS software (version 16) and expressed as means ± SEM. The differences between the groups were assessed using analysis of variance (ANOVA). Differences were considered significant if the calculated p-values were less than 0.05.

## Results

### Inhibition of cell viability in response to TQ treatment

**Figure 1:**
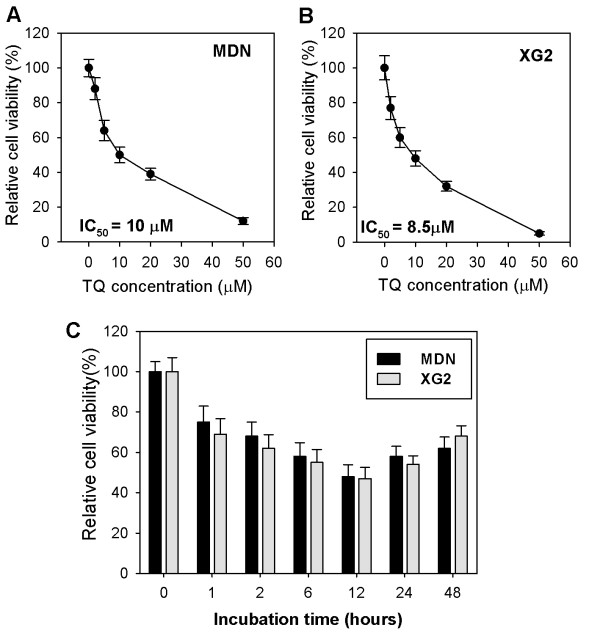
**Dose and time responses of cell death after TQ treatment**. Five thousand MDN (**A**) and XG2 (**B**) cells, per well were seeded in triplicate onto 96-well plates; treated with different concentrations of TQ at 0, 1, 2, 5, 10, 20 and 50 μM for overnight; measured cell viability by the MTT method and presented as percent cell viability. (**C**) the cells were treated for different incubation hours with the representative IC50 of TQ. Data are the representative of three independent experiments, and are presented as the mean percentage of viable cells ± SEM.

We first investigated the ability of TQ to induce growth arrest in the MM cell lines MDN and XG2. These cells were treated overnight with different doses of TQ, and the cytotoxic effect of TQ was measured using the MTT uptake method. We found that TQ induced growth arrest in these cells in a dose-dependent manner, with IC_50 _values of 10 μM for MDN (Figure 1 A) and 8.5 μM for XG2 (Figure 1B; *p *< 0.05; n = 3). Furthermore, TQ significantly inhibited the growth of MDN and XG2 cells in a time-dependent manner. This effect was apparent after incubation for 2 hours, peaked after 12 h and decreased thereafter (Figure 1C; *p *< 0.05; n = 3).

### TQ inhibits CXCL-12-mediated actin polymerization

**Figure 2:**
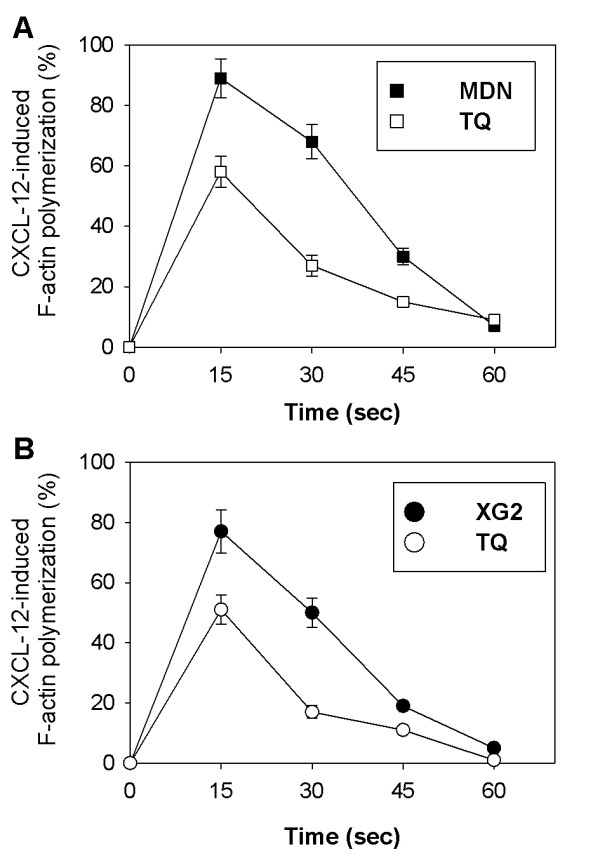
**Effects of TQ on CXCL-12-mediated F-actin polymerization**. MDN (**A**) and XG2 (**B**) cells were untreated (open symbols) or treated with TQ for 12 hr (black symbols). F-actin polymerization in response to CXCL-12 was measured. The results are expressed as the percentage change in MFI (n = 5) ± SEM, as described in the *Materials and Methods *section.

Actin and microtubules provide a dynamic cellular framework to orchestrate and ultimately control cellular activation and cancer metastasis. Therefore, we monitored actin polymerization in response to CXCL-12 stimulation in MDN and XG2 cells. The cells were cultured for 12 hours in the presence or absence of TQ prior to stimulation with CXCL-12 (250 ng/ml) every 15 sec. The degree of F-actin polymerization was determined using flow cytometry. In untreated control MDN cells (closed squares), the percentage of F-actin polymerization was 89 ± 6.4%, 68 ± 5.6%, 30 ± 2.8% and 7 ± 1% after 15, 30, 45 and 60 sec, respectively. However, the percentage of F-actin polymerization was significantly reduced to 58 ± 5.1%, 27 ± 3.5%, 15 ± 1.3% and 9 ± 0.85% after 15, 30, 45 and 60 sec, respectively, in TQ-treated cells (open squares, Figure 2A; *p *< 0.05; n = 5). Similarly, the percentage of F-actin polymerization in untreated control XG2 cells (closed circles) was 77 ± 7.1%, 50 ± 4.8%, 19 ± 1.5% and 5 ± 0.7% after 15, 30, 45 and 60 sec, respectively. However, in TQ-treated cells, the percentage of F-actin polymerization was significantly reduced to 51 ± 4.9%, 17 ± 2.1%, 11 ± 1% and 1 ± 0.8% after 15, 30, 45 and 60 sec, respectively (open circles, Figure 2B; *p *< 0.05; n = 5).

### TQ inhibits IL-6-induced proliferation in MM cells

**Figure 3:**
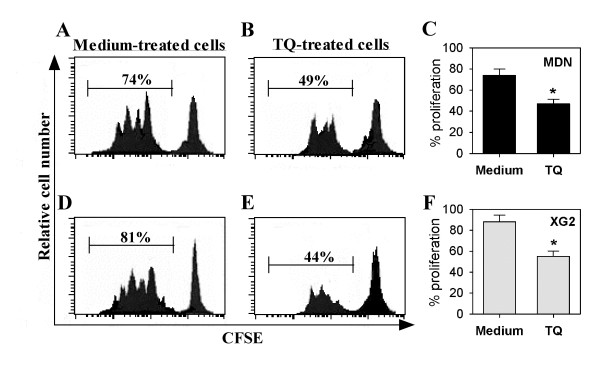
**TQ diminishes IL-6-induced proliferation of MM cells**. The ability of MDN (**A**, **B**, **C**) and XG2 (**A**, **E**, **F**) cells to proliferate in the presence or absence of TQ was evaluated using CFSE assays and flow cytometry. CFSE-labeled cells were then left untreated (medium-treated cells) (**A, D**) or treated with TQ (**B, E**). One representative experiment of five showing that histograms were gated on viable cells, and the mean of the left histograms represents the percentage of CFSE-lo (proliferating) cells. Combined data from 5 different experiments are expressed as the mean percentage of proliferating MDN (**C**) and XG2 (**F**) cells ± SEM, * p < 0.05.

IL-6 plays a major role in the development and maintenance of MDN and XG2 cells. Therefore, we monitored the effects of TQ on IL-6-mediated MDN and XG2 cell proliferation using a CFSE dilution assay and flow cytometry. As shown in Figure 3, the percentage of proliferating MDN cells was markedly reduced from 74% in the medium-treated cells (Figure 3A) to 49% in the TQ-treated cells (Figure 3B). Similarly, the percentage of proliferating XG2 cells was markedly reduced from 81% in the medium-treated cells (Figure 3D) to 44% in the TQ-treated cells (Figure 3E). Our data revealed that the proliferative capacity of MDN cells was significantly reduced from 73 ± 5.8% in the medium-treated cells to 47 ± 4.4% in the TQ-treated cells (Figure 3C; *p *< 0.05; n = 5). Similarly, TQ treatment significantly reduced IL-6-mediated XG2 cell proliferation from 86 ± 6.4% in the medium-treated cells to 52 ± 5.1% in the TQ-treated cells (Figure 3F; *p *< 0.05; n = 5).

### TQ suppresses phosphorylation of STAT3 and its downstream signaling effectors Bcl-2 and Bcl-_XL_

**Figure 4:**
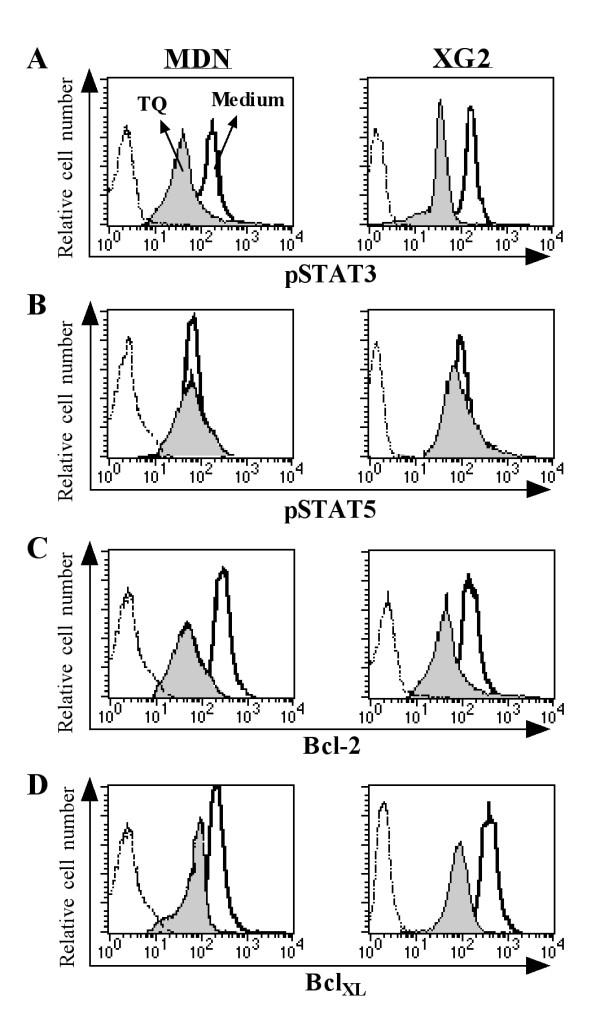
**Flow cytometry quantification of phospho-STAT3, phospho-STAT5 and expression of Bcl-2/Bcl-_XL _after TQ treatment**. Phosphorylation of STAT3 (**A**) and STAT5 (**B**) as well as the anti-apoptotic molecules Bcl-2 (**C**) and Bcl-_XL _(**D**) in MDN (left panel) and XG2 (right panel) cells were monitored after 12-hr incubation in the presence or absence of TQ using Phosflow mAbs and flow cytometric analysis. Histograms for medium-treated cells are displayed as open bold line histograms, as gray-filled histograms in TQ-treated cells, while the isotype controls are represented by open dotted thin line histograms. One representative experiment of three in shown.

We investigated whether TQ treatment impairs signaling by STAT3, a signaling transcription factor important for MM cell maintenance and survival. Using Phosflow assays and flow cytometry, we found that TQ markedly decreased STAT3 phosphorylation (Figure 4A) but had no effect on STAT5 phosphorylation (Figure 4B). STAT3 regulates several anti-apoptotic proteins, including Bcl-2, Bcl-_XL _and survivin. We also found that TQ decreases Bcl-2 (Figure 4C) and Bcl-_XL _(Figure 4D) expression.

**Figure 5:**
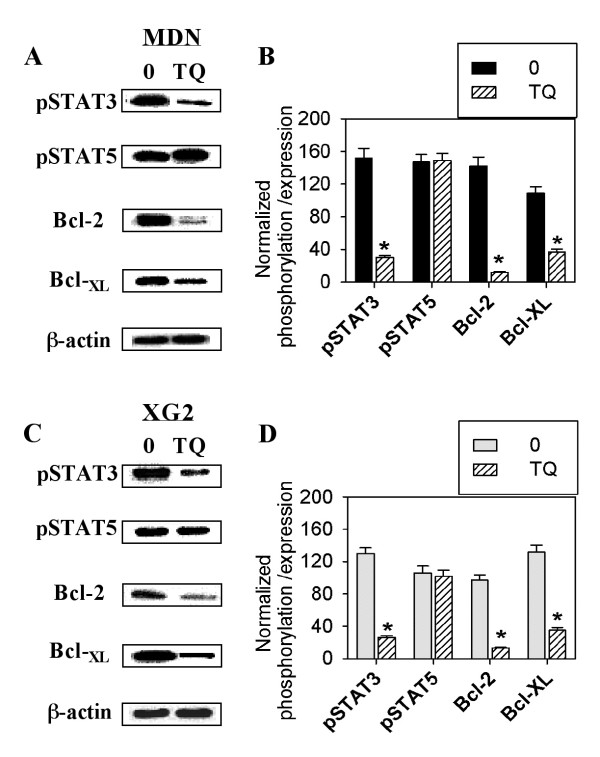
**Western blot analysis of phospho-STAT3, phospho-STAT5 and expression of Bcl-2/Bcl-_XL _after TQ treatment**. Western blot analysis for the phosphorylation of STAT3, STAT5 and the expression of Bcl-2 and Bcl-_XL _was conducted in MM cells after 12-hour treatment with TQ. Protein bands from one representative experiment out of the five performed are shown for MDN (**A**) and XG2 (**C**). The phosphorylation of STAT3, STAT5 and the expression of Bcl-2 and Bcl-_XL _was normalized to β-actin on stripped blot. Combined data from 5 different experiments are expressed as the mean ± SEM of normalized protein values in MDN (**C**) and XG2 (**F**) cells after TQ treatment (hatched bars), * p < 0.05.

Because antibodies directed against phospho-proteins vary in their sensitivities depending on the technique, we confirmed our results using antibodies against pSTAT3, pSTAT5, Bcl-2 and Bcl-_XL _and western blotting. Figures 5A and 5C show the data obtained in one representative experiment out of five experiments performed in MDN and XG2 cells. The levels of phosphorylated STAT3 and STAT5 and the expression of Bcl-2 and Bcl-_XL _were normalized to the amount of total β-actin in five separate experiments. In MDN cells (Figure 5B), STAT3 phosphorylation and the expression of Bcl-2 and Bcl-_XL _were significantly diminished from 152 ± 11.7%, 142 ± 11.3% and 109 ± 7.9% in untreated cells to 30 ± 2.8%, 12 ± 1.1% and 37 ± 3.3% in TQ-treated cells, respectively (*p *< 0.05; n = 5). Although STAT5 was clearly phosphorylated in both TQ-treated and untreated cells, TQ had no effect on STAT5 phosphorylation. Similarly, STAT3 phosphorylation and the expression of Bcl-2 and Bcl-_XL _were significantly reduced from 130 ± 7.1%, 97 ± 6.7% and 132 ± 8.2% in untreated XG2 cells to 26 ± 2.5%, 13 ± 1.1% and 35 ± 3.4% in TQ-treated XG2 cells, respectively (Figure 5D; *p *< 0.05; n = 5).

## Discussion

Although the anti-tumor and cytotoxic effects of TQ on numerous cancer cells have been known for many years [14,22-24], little is known regarding its effects on MM cells. Here, we investigated the effects of TQ on two different MM cell lines (MDN and XG2). Proliferation and survival are critically important for tumor growth and metastatic spreading; therefore, proliferation and survival have been attractive targets for tumor therapy. First, we assessed the ability of TQ to induce growth arrest in the MM cell lines MDN and XG2, and we found that TQ induces growth arrest in both MDN and XG2 cells in a dose- and time-dependent manner. Our results agree with the results of Shoieb et al., [2003] who attributed this growth inhibition to apoptosis and cell cycle arrest [15]. Cell migration is a critical step in tumor invasion and metastasis, and regulation of this process may lead to appropriate therapies for treating cancer. Actin cytoskeletal reorganization is the primary mechanism of cell motility and is essential for most types of cancer cell migration [25]. Therefore, we monitored actin polymerization in response to CXCL-12 stimulation and found that TQ decreases CXCL-12-mediated actin polymerization in MDN and XG2 cells. Indeed, previous studies have suggested the involvement of CXCL12/CXCR4 in the maintenance and survival of MM cells both in vivo and in vitro [6,26].

IL-6 is a pleiotropic cytokine with biological activities in a wide variety of cells. Normally, IL-6 induces B-cell differentiation, but in myeloma it induces proliferation and inhibits apoptosis [27]. IL-6 is especially involved in malignant plasma cell expansion. IL-6, which is derived from both autocrine and paracrine sources, is the key growth and survival factor for MM cells [28,29]. Therefore, we investigated the effects of TQ on IL-6-mediated MDN and XG2 cell proliferation using a CFSE dilution assay and flow cytometry. We found that TQ inhibits IL-6-induced proliferation of MM cells, which agrees with previous results of Shoieb et al., [2003] who reported that TQ inhibits tumor cell proliferation through a mechanism that involves cytotoxicity [15]. In addition, previous studies have shown that TQ inhibits cell proliferation in many types of cancer cells [14]. Recently, Li et al., found that TQ inhibits MM cell proliferation by suppressing the signal transducer and activator of transcription 3 activation pathway [18].

Members of the STAT family of transcription factors regulate the expression of gene products involved in cell survival, proliferation, chemoresistance, and angiogenesis [7]. Previous studies have reported that STAT3 is constitutively activated in a growing number of tumor-derived cell lines and samples from human cancers, including lymphomas and leukemias [8]. Furthermore, a constitutively active STAT3 mutant has been shown to induce cellular transformation in fibroblasts, which reveals its oncogenic potential [30]. In MM cells, STAT3 signaling has also been reported to play an important role in cell maintenance and survival [31]. Our data demonstrate that TQ suppresses STAT3 phosphorylation and the expression of its downstream signaling effectors Bcl-2 and Bcl-_XL _but has no effect on STAT5 phosphorylation. Our results agree with those of Li et al., [2010] who found that TQ down-regulates the expression of STAT3-regulated gene products, such as cyclin D1, Bcl-2, Bcl-_XL_, survivin, Mcl-1 and vascular endothelial growth factor [18].

## Conclusions

Our data reveal the anti-tumor effects of TQ. Therefore, TQ administration is a new approach that can enhance immunogenicity, reduce cell proliferation and increase apoptosis in cancer cells by suppressing STAT3 phosphorylation and Bcl-2/Bcl-_XL _expression.

## List of abbreviations

Bcl-2: B-cell lymphoma 2; BM: bone marrow; CFSE: carboxyfluorescein diacetate succinimidyl ester; MM: multiple myeloma; STAT: signal transducer and activator of transcription; TQ: thymoquinone.

## Competing interests

All authors have read and agreed the contents of the manuscript and approved the submission. The authors declare no conflicts of interest, state that the manuscript has not been published or submitted elsewhere, state that the work complies with Ethical Policies of the Journal and the work has been conducted under internationally accepted ethical standards after relevant ethical review.

## Authors' contributions

GB carried out the immunology studies, participated in the study design and drafted the manuscript and preparing the figures. MM carried out the immunoassays and participated in the figures preparation. FA participated in the design of the study and coordination and helped to draft the manuscript. All authors read and approved the final manuscript.
